# X-linked Alport syndrome: pathogenic variant features and further auditory genotype-phenotype correlations in males

**DOI:** 10.1186/s13023-018-0974-4

**Published:** 2018-12-22

**Authors:** Xiao Zhang, Yanqin Zhang, Yanmei Zhang, Hongbo Gu, Zhe Chen, Lei Ren, Xingxing Lu, Li Chen, Fang Wang, Yuhe Liu, Jie Ding

**Affiliations:** 1 Department of Otolaryngology, Head and Neck Surgery, Peking University First Hospital, Beijing, 100034 China; 2 Department of Pediatrics, Peking University First Hospital, Beijing, 100034 China

**Keywords:** X-linked Alport syndrome, Hearing features, Gene mutation

## Abstract

**Objective:**

To analyze the clinical audiological characteristics of X-Linked Alport syndrome (XLAS) in males and their relationships with genotypes.

**Methods:**

The clinical data of 87 male patients with AS were reviewed. Hearing levels were evaluated using pure tone audiometry (PTA) testing, acoustic immittance, and otoacoustic emissions (OAE) testing. The genotypes of *COL4A5* and the pathogenic variants were analyzed. The relationships between auditory phenotypes and genotypes were analyzed.

**Results:**

Among the 87 patients, the number of patients with normal hearing and hearing loss were 32 and 55, respectively. In all cases, the hearing loss was characterized as bilateral symmetrical sensorineural deafness. Majority of the patients had mild-to-moderate hearing loss. Hearing loss usually started in the middle frequency range and gradually affected high frequencies, at school age and gradually increased with increasing age. However, it maintained a relatively steady level of 50–60 dB HL during the teenage years. The audiometric curves included groove-type in 51 cases (92.73%). Patients were identified to have 60 different *COL4A5* pathogenic variants. Of the 49 patients who were followed-up for more than 2 years, 28 cases presented a decreasing trend in the hearing level of about 5 dB per year. The degree of hearing loss was positively correlated with gene mutation type and renal function.

**Conclusions:**

Hearing loss in males with XLAS is symmetrical sensorineural, and progressive with increasing age. There is a significant correlation between the degree of hearing loss and genotype, renal function, and age.

## Background

Alport syndrome (AS) is a rare hereditary disease with an incidence of 1/5000–1/50000, involving the basement membranes of the kidneys, ears and eyes. It is characterized by chronic kidney disease, sensorineural hearing loss, and ocular abnormalities [1], affecting at least 1 in 50,000 individuals [2]. There are three inheritance patterns: X-linked dominant inheritance of *COL4A5* mutations, autosomal recessive of *COL4A3* or *COL4A4* mutations, and autosomal dominant of *COL4A3* or *COL4A4* mutations, with a percentage of 80, 15, and 5%, respectively [3, 4]. The *COL4A5* and *COL4A3/COL4A4* genes code for different collagen type IV chains, including α3, α4 and α5 chains, that form heterotrimers in the basement membranes [5].

Type IV collagen is the main collagen component of the basement membrane, playing an important role in the functioning of basement membrane barrier, and contains six kinds of alpha chains (α1–6) [6–8]. Defects in α3, α4 and α5(IV) chains can cause abnormal formation of mature alpha chains, further disrupting the formation of type IV collagen molecules and changing the structure of the basement membrane. These physiological changes finally results in the dysfunction of organs such as the glomerulus, the retina and the cochlea.

Each alpha chain of type IV collagen contains both collagen and non-collagen regions [9]. The collagen region is rich in the triple structure of glycine (Gly)-X-Y (X, Y for any amino acid) [5], and glycine is necessary in the Gly-X-Y triple structure of the collagen domain. It is the only amino acid that is small enough to enter the helix center of collagen.

Earlier studies [10–13] have revealed that hearing loss is a result of alterations of basement membranes in AS patients. The α5(IV) chain is present in the basement membrane and spiral ligament of the whole Corti organ, and the results of immunostaining of the cochlea for antibodies against α5(IV) chain of type IV collagen has been reported [7]. Mutations in *COL4A5* gene resulted in the abnormality or absence of the corresponding coding product. Therefore, the normal IV collagen network structure cannot be constructed, resulting in basement membrane injury and hearing loss [8, 14]. Different variant types such as deletions, insertions, splicing variants, direct or indirect nonsense mutations, and missense variants (including Gly or non-Gly substitutions) in *COL4A5* gene have been identified [3].

Genotype–phenotype correlations in the kidney have been studied for X-linked AS disease [15, 16]; however, there are fewer studies regarding the human inner ear in AS. Several studies have reported that hearing loss is one of the early signs of AS [17–19]. It has become evident that hearing loss appears at the end of childhood or at the beginning of adolescence in boys with X-linked disease. A continuous progression in hearing loss indicates a poor prognosis [13]. Most of the reports have shown that hearing loss in AS is sensorineural, progressive and symmetrical [20], often affecting the middle but especially high frequencies in a moderate or severe extent. The groove-type audiometric curve is present in only about 47.1% of AS patients [20]. Hearing loss related to X-linked AS mainly occurs in male patients, and the level of hearing loss is always more severe in men than in women [21]. It has been reported that the risk of developing hearing loss before the age of 30 was about 60% in patients with a missense mutation, whereas it reached 90% for patients with other types of mutations including splice site mutations [16].

Till date, there is no trend in hearing changes or possible influencing factors on the hearing level have been reported. Therefore, this study aimed to explore the clinical audiological characteristics in male patients with AS and the auditory genotype–phenotype correlations. The results of this study might be useful in predicting the trends in hearing changes and in providing timely interventions for such patients.

## Materials and methods

### Study population

Eighty-seven patients diagnosed with X-linked Alport syndrome (XLAS), with an age range of 2–33 years and a median age of 13 years were studied between March 2013 and March 2017. They were divided into 6 different age groups: pre-school age (< 6 years old; n_patients_ = 16, n_ears tested_ = 32), school-age children (7~9 years old; n_patients_ = 22, n_ears tested_ = 44), school-age children (10~12 years old; n_patients_ = 20, n_ears tested_ = 40), teenage children (13~15 years old, n_patients_ = 11, n_ears tested_ = 22), teenage children (16~18 years old, n_patients_ = 11, n_ears tested_ = 22), and adults (≥18 years old, n_patients_ = 7, n_ears tested_ = 14). None of the patients had end-stage renal disease. Diagnosis of X-linked AS is highly possible if glomerular hematuria coincides with one of the following criteria: (i) a family history of AS, and no other cause of hematuria, (ii) characteristic clinical features, including lenticonus or retinopathy, and (iii) lack of expression of the collagen α5(IV) chains in the glomerular basement membrane (GBM) or epidermal basement membrane (EBM). The diagnosis is confirmed if there is a lamellated GBM or a pathogenic mutation in the *COL4A5* gene [2, 22].

The identified variants were checked in the following databases: NCBI dbSNP build 141 (http://www.ncbi.nlm.nih.gov/SNP/), 1000 Genomes Project (http://www.1000genomes.org/), Exome sequencing project (ESP6500. http://evs.gs.washington.edu/EVS/), Exome Aggregation Consortium (ExAC) (http://exac.broadinstitute.org/) and BGI in-house database. SIFT, PolyPhen 2 and MutationTaster and Human Splicing Finder were used. The pathogenicity of variants identified was assessed according to the ACMG (American College of Medical Genetics and Genomics) standards [23, 24]. The diseases present were clinically diagnosed by kidney specialists and analyzed the inheritance patterns. This study has been approved by the hospital ethics committee, and all parents of children undergoing mutation testing signed informed consent forms.

The data collected included age, variant types, urinary protein/24 h (Up/24 h), and renal function (Creatinine clearance rate, Ccr). The time interval between the hearing test, the renal function test, and the urine protein test did not exceed 3 months.

### Gene amplification and DNA sequencing

Total RNA were extracted from skin fibroblasts and analyzed according to the previous report [24]. Genomic DNA were extracted from peripheral blood leukocytes using a FlexiGene DNA Kit (Qiagen) according to the manufacturer’s protocol. Geomic DNA from probands was screened for mutations in *COL4A3–6* genes. Targeted next generation sequencing (NGS) was performed by BGI-Tianjin, China as published before. All pathogenic mutations identified with NGS were confirmed by Sanger sequencing [25].

Mutations of *COL4A5* were divided into two types: missense mutations (including Gly and non-Gly substitutions) and other mutations (deletions, insertions, splicing variants, and nonsense mutations). The relationships between audiological phenotype and mutation of *COL4A5* gene were analyzed.

### Otolaryngological physical examination

All patients underwent a normal otolaryngological physical examination. Audiological testing including pure tone audiometry (PTA) testing, acoustic immittance and otoacoustic emissions (OAE) testing were performed. OAE testing was conducted in 4 children who did not cooperate with PTA due to young age.

#### Outcome classification

To observe the characteristics of audiological curves of hearing loss in these patients, the average hearing thresholds (pure tone audiometry, PTA’) at 500, 1000, and 2000 Hz were taken in pure tone audiometry for statistical analysis. The degree of deafness was referred to the World Health Organization (WHO) 1997 criterion, wherein the PTA was the average hearing threshold at 500, 1000, 2000 and 4000 Hz. The hearing loss was divided into four levels, mild (26–40 dB HL), moderate (41–60 dB HL), severe (61–80 dB HL), and profound (≥81 dB HL). Symmetrical hearing loss was present if the difference in hearing loss at the same frequency in both ears was less than 15 dB. The audiometric curves included ascending-type, groove-type, descending-type, flat-type, and high frequency steep drop-type [26]. 0.25 kHz and 0.5 kHz are considered as low frequencies, 1 kHz and 2 kHz are middle frequencies, and 4 kHz and8kHz are high frequencies.

### Statistical analysis

Statistical analysis was performed using SPSS 20.0 (SPSS Inc., Armonk, NY, USA). All continuous data were displayed as means ± standard deviation. The unpaired Student’s t-test was used to compare the quantitative variables among different groups. Pearson’s chi-squared test was used to compare the categorical variables among different groups. Multiple logistic regression analysis was used to evaluate the significance of independent variables. *P* < 0.05 was considered to be statistically significant.

## Results

### Clinical audiological characteristics

**Fig. 1:**
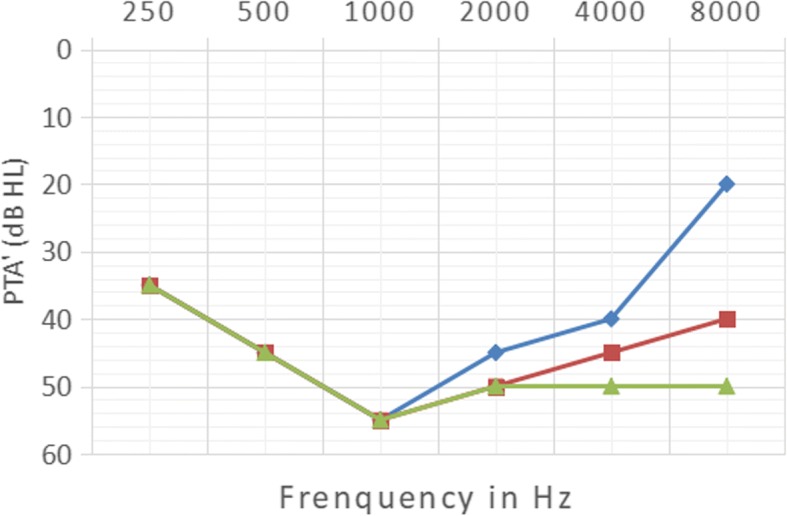
Continuous changes of hearing in a child for 3 years.(blue line: 2015(8-year-old), red line: 2016(9-year-old), green line: 2017(10-year-old))

Among the 87 patients, 83 received pure tone audiometry tests and 4 patients (2–3 years old) received OAE tests. All patients received acoustic impedance tests. The results of OAE testing were normal. The acoustic immittance of all patients was type A. The number of patients with normal and abnormal hearing was 32 and 55, respectively, with an incidence of hearing loss of 63.2% (55/87). The auditory results were characterized by bilateral symmetrical sensorineural deafness. Majority of the patients presented with mild to moderate hearing loss. The hearing loss was usually started in the middle frequency range and gradually affected the higher frequencies (Fig. 1). In 55 patients with hearing loss, the distribution of audiometric curves was: groove-type in 51 cases (92.73%) (Fig. 1, blue line), flat-type in 2 cases, ascending-type in 1 case, and descending-type in 1 case.

#### The relationship between PTA’ and age

A total of 87 children were followed up for 5 years, and 175 audiometric data were obtained. We analyzed the relationship between hearing and corresponding age.

**Fig. 2:**
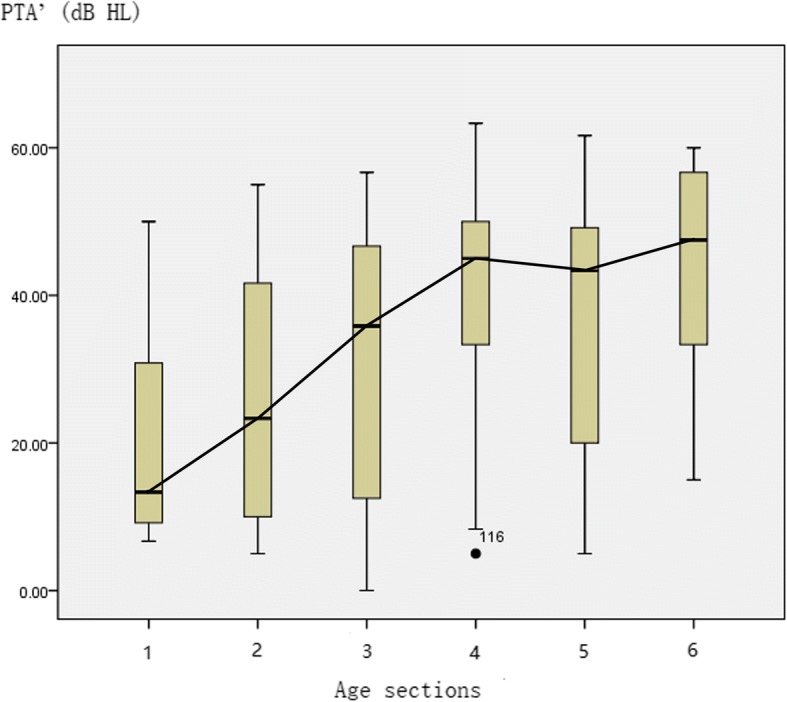
The figure represents the median and interquartile range of hearing for different age groups. Relationship between PTA’ and age group: X-axis represents the age group: gp 1: ≤6 years (n_ears_ = 15); gp 2: 7–9 years (n_ears_ = 43); gp 3: 10–12 years (n_ears_ = 52); gp 4: 13–15 years (n_ears_ = 30); gp 5: 16–18 years (n_ears_ = 19); and gp 6: > 18 years (n_ears_ = 16); Y-axis represents the PTA’ (dB HL); PTA’: mean of hearing thresholds at 0.5 kHz, 1 kHz, and 2 kHz; *n* = 175 data points

Hearing was normal before school age. Hearing loss usually started at school age and was gradually increased with increasing age. However, it maintained a relatively steady platform level during the teenage years (13–18 years) with a value of 50–60 dB HL (Fig. 2). These results concluded that hearing level was declined with increasing age.

#### The relationship between hearing threshold at 1 kHz and age

**Fig. 3:**
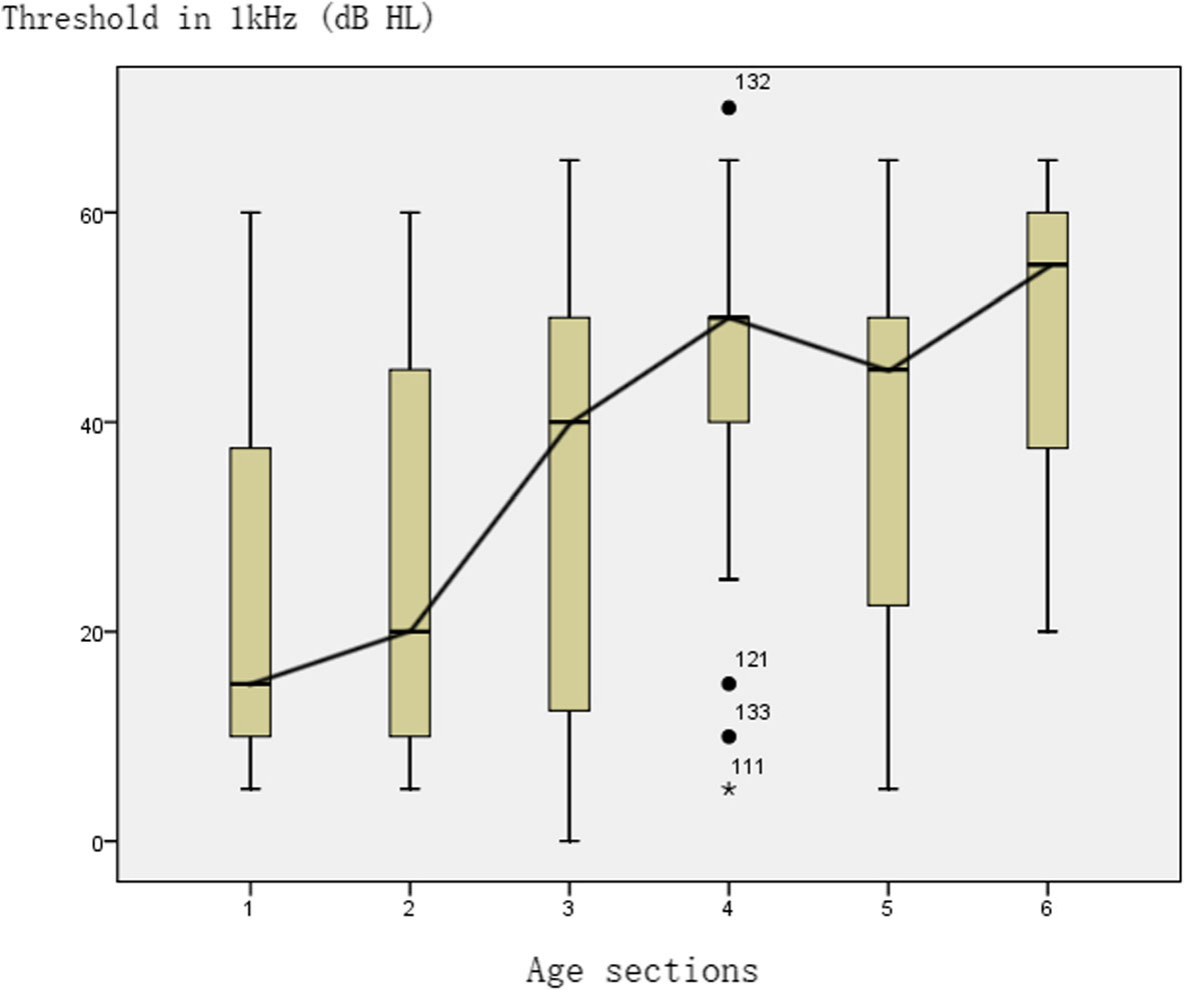
The figure represents the median and interquartile range of hearing threshold at 1 kHz for different age groups. Relationship between hearing threshold at 1 kHz and age. X-axis represents the age group: gp 1: ≤6 years (nears = 15); gp 2: 7–9 years (nears = 43); gp 3: 10–12 years (nears = 52); gp 4: 13–15 years (nears = 30); gp 5: 16–18 years (nears = 19); and gp 6: > 18 years (nears = 16); Y-axis represents the hearing threshold at 1 kHz; n = 175 data points

Next, we analyzed the relationship between hearing threshold and age at a single frequency (1 kHz and 2 kHz). The relationship between hearing and age of 1 k Hz was shown in Fig. 3 (follow-up of 5 years, and a total of 175 audiometric data).

Hearing loss usually started at school age and was gradually increased with increasing age. Initially, hearing level dropped gradually and then more rapidly, reaching a platform period during the teenage years (13–18 years old) with a level of 50–60 dB HL. After the teenage years, the hearing level was continued to decrease with increasing age. These results suggested that the hearing level was declined with increasing age.

The relationship between hearing threshold at 2 kHz and age was analyzed. We found that the hearing loss at 2 kHz was earlier than at 1 kHz, which then gradually reached 50 dB HL level in the platform period, and then continued to increase with increasing age.

We concluded that the hearing changes with age at 1 kHz and 2 kHz are similar to the changes in PTA’.

### *COL4A5* pathogenic variants

All 87 patients were tested for *COL4A5/6* genotype, where 60 different *COL4A5* pathogenic variants were identified in 63 patients.

In this study, 17 gene variants (Table 1) have already been reported for their pathogenicity in the previous studies. Among the remaining unreported gene pathogenic variants, there were 16 indel mutations, 5 splicing variants, 2 nonsense mutations, and 20 missense variants. Of the 20 unreported missense variants, 14 were glycine substitutions and 6 were non-glycine substitutions. The conservation analysis of amino acids was carried out by Genomic Evolutionary Rate Profiling (GERP) and PhyloP software. Polyphen-2 and SIFT gene prediction software were used to predict the possibility of pathogenicity of these 20 unreported missense variants (Table 2). Mutation Taster software was used to predict the possibility of pathogenicity of these severe variants (indels, splicing and nonsense mutations).

**Table 1:** Genetic variant test results

Type of pathogenic variant (mutation)	No. of patients (%) (*n* = 63)	Hearing	Base or amino acid changes (N: number of patients; n: number of variants)
Missense	Replacement of glycine 25 (39.68%)	Normal	(*N* = 17; *n* = 17) c.3080G > T^*^(p.Gly1027Val);c.2723G > A^*^(p.Gly908Glu);c.395G > A(p.Gly132Glu);c.3383G > A(p.Gly1128Asp);c.892G > C(p.Gly298Arg);c.3508G > A^*^(p.Gly1170Ser);c.2714G > A^*^(p.Gly905Asp);c.2858G > T(p.Gly953Val);c.1175G > A(p.Gly325Arg);c.3418G > C(p.Gly1140Arg);c.1121G > T(p.Gly374Val);c.3319G > A^*^(p.Gly1107Arg);c.2287G > A^*^(p.Gly763Arg);c.3446G > T(p.Gly1149Val);c.3704G > C(p.Gly1235Ala);c.1861G > T^*^(p.Gly621Cys);c.2509G > A^*^(p.Gly837Ser)
		Abnormal	Mild(*N* = 2; *n* = 2): c.2605G > A^*^(p.Gly869Arg);c.1410G > T(p.Gly403Val)
			Moderate(*N* = 6; *n* = 5): c.4787G > A(p.Gly1596Asp);c.3704G > A(p.Gly1235Asp);c.1940G > T(p.Gly647Val);c.2678G > T^*^(p.Gly893Val);c.3170G > A(p.Gly1057Glu)
	Replacement of non-glycine 7 (11.11%)	Normal	(*N* = 5; *n* = 4): c.4688G > A(p.Arg1563Gln);c.3713G > A(p.Ala1238Thr);c.4862 T > C(p.Leu1621Ser);c.4427G > T(p.Cys1476Phe)
		Abnormal	Moderate(*N* = 2, *n* = 2): c.4186C > T(p.Pro1396Ser);c.1984G > A(p.Ser992Asn)
Rearrangement	9 (14.29%)	Normal	(*N* = 2; *n* = 2): Exon25del^*^ c.1484-1501delAACCTGGTTTGCCAGGTC(p.Gln495Glyfs*6)
		Abnormal	Mild(*N* = 2; *n* = 2): Exon4-37del; Exon45del
			Moderate (*N* = 5; *n* = 4): c.3816-3823delAGGTCTCC(p.Gly1273Trpfs*6);c.1106-1110delAA(p.Lys370Argfs*40); E42Del;COL4A5E1 + E2-COL4A6E1 + E2 Del; Exon48-50Del
Splice	7 (11.11%)	Normal	(*N* = 1; *n* = 1): c.456 + 2 T > C
		Abnormal	Moderate(*N* = 5; *n* = 5): c.(781-7 T > A); c.2042-2A > G; c.IVS24 + 1G > A; c.4995-2A > G^*^; c.2245-2A > G^*^
			Severe(*N* = 1; *n* = 1): c.937 + 5G > A
Nonsense	5 (7.94%)	Normal	(*N* = 1; *n* = 1): c.1117C > T^*^(p.Arg373*)
		Abnormal	Moderate(*N* = 3; *n* = 3): c.3850A > T(p.Lys1284*);c.5038C > T^*^(p.Arg1680*);c.107C > G^*^(p.Ser36*)
			Severe(*N* = 1; *n* = 1): c.4078G > T(p.G1360*)
Frameshift	10 (15.87%)	Normal	(*N* = 1; *n* = 1): c.2842DelC(p.Leu948Phefs*48)
		Abnormal	Mild(*N* = 1; *n* = 1): c.1486-1491insG(p.Pro446*)
			Moderate(*N* = 8; *n* = 7): c.3745delG^*^(p.Gly1249*);c.2471_2473insA(p.Gly825Argfs*30);c.2566_2567delC(p.Pro856Glnfs*18);c.660_662delA(p.Asn221Ilefs*33);c.2080delA(p.Ile694Tryfs*42);c.4782delG(p.Trp1549*);c.1269delA(p.Asp425Tyrfs*49);

Note: Gene variants marked with an asterisk (*) indicate 17 gene variants that have already been reported for their pathogenicity

The accession number for the reference isoform of the COL4A5 gene is NM_033380.2

The mutation that the upper right-hand corner is marked by“*“indicates that the gene mutation has been reported

**Table 2:** Pathogenicity prediction for 20 unreported missense variants in *COL4A5*

Gene	Variant	Polyphen-2 score		SIFT^3^	GERP++RS^4^	phyloP100way^e^ vertebrate	PhyloP20way^6^ mammalian
		Polyphen-2 HDIV^1^	Polyphen-2 HVAR^2^				
*COL4A5*	c.395 G > A, p.Gly132Glu	1	1	0	6.13	8.383000	1.048000
*COL4A5*	c.3383G > A, p.Gly1128Asp	1	1	0.01	5.45	7.901000	1.048000
*COL4A5*	c.892G > C, p.Gly298Arg	1	1	0	5.5	8.333000	1.048000
*COL4A5*	c.2858G > T, p.Gly953Val	1	1	0	5.97	7.247000	1.048000
*COL4A5*	c.1175G>A, p.Gly325Arg	1	1	0	unknown	unknown	unknown
*COL4A5*	c.2858G > T, p.Gly953Val	1	1	0	5.97	7.247000	1.048000
*COL4A5*	c.3418G>C, p.Gly1140Arg	1	1	0	5.45	6.217000	1.048000
*COL4A5*	c.4688G > A, p.Arg1563Gln	1	1	0	unknown	unknown	unknown
*COL4A5*	c.3713G > A, p. Ala1238Thr	0.996	0.885	0	5.97	7.155000	1.048000
*COL4A5*	c.1121G > T, p.Gly374Val	1	1	0.13	5.2	7.122000	0.998000
*COL4A5*	c.4862 T > C, p.Leu1621Ser	1	1	0	unknown	unknown	unknown
*COL4A5*	c.3446G > T, Gly1149Val	unknown	unknown	0	Unknown	unknown	unknown
*COL4A5*	c.3704G > C, p.Gly1235Ala	0.969	0.69	0.1	5.97	9.459000	1.048000
*COL4A5*	c.4427G>T, p.Cys1476Phe	1	1	0	unknown	unknown	unknown
COL4A5*	c.1410G > T, p.Gly403Val	1	1	0	unknown	unknown	unknown
*COL4A5*	c.4186C > T, p.Pro1396Ser	unknown	unknown	0.23	4.14	1.857000	0.935000
COL4A5*	c.4787G>A, p.Gly1596Asp	1	1	0	5.57	10.003000	1.048000
COL4A5*	c.3704G>A, p.Gly1235Asp	0.999	0.986	0	5.97	9.459000	1.048000
*COL4A5*	c.1984G>A, p.Ser992Asn	1	1	0.01	unknown	unknown	unknown
COL4A5*	c.3170G > A, p.Gly1057Glu	1	1	0	5.76	7.136000	1.048000

The accession number for the reference isoform of the COL4A5 gene is NM_033380.2

The *COL4A5* is marked by“*“indicates the Gly substitutions that result in severe disease

Note:

^1^Scores of 0.0–0.446 (benign), 0.447–0.909 (possibly damaging), and 0.909–1 (probably damaging)

^2^Scores of 0.0–0.446 (benign), 0.447–0.909 (possibly damaging), and 0.909–1 (probably damaging)

^3^Scores of 0.00–0.05 = D (damaging), 0.06–1.0 = T (tolerated)

^4^The larger the score, the more conserved the site. Scores ranged from −12.3 to 6.17

^5^Phylop (phylogenetic p-values) conservation score based on multiple alignments of 100 vertebrate genomes (including human). Scores ranged from −20.0 to 10.003 in dbNSFP

^6^Phylop (phylogenetic *p*-values) conservation score based on multiple alignments of 20 mammalian genomes (including human). Scores ranged from −13.282 to 1.199 in dbNSFP

We studied the pathogenicity of the 43 unreported variants, and it can be seen that the unreported gene variants in this study are basically pathogenic, and these nucleic acid sequences are highly conserved.

### Correlations between genotypes and phenotypes

#### The relationship between PTA’, age and mutation

Among the 87 children, 63 children were identified to have clear gene mutations. Of the 32 patients with missense pathogenic variants, 9 presented hearing loss (28.1%). Of the 31 patients with indels, splicing, or nonsense mutations, 26 presented hearing loss (83.9%). Of the 55 patients, 35 patients with hearing loss presented gene pathogenic variants, and 26 with severe pathogenic variants (26/35; 74.3%).

**Fig. 4:**
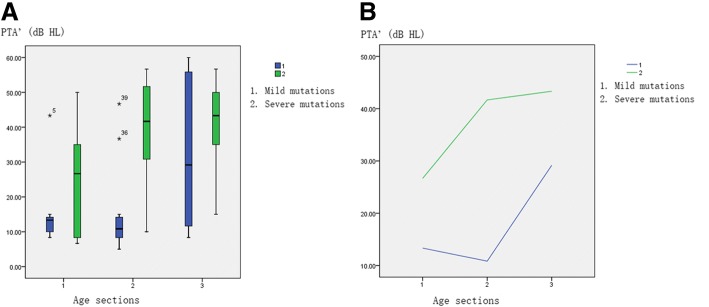
**a, b** Relationship between PTA’, age and mutation. X-axis represents age group: gp 1: ≤6 years old (*n* = 15); gp 2: 7–12 years old (*n* = 31); and gp 3: ≥13 years old (*n* = 16); Y-axis represents PTA’ (mean of hearing thresholds at 0.5 kHz, 1 kHz, and 2 kHz); *n* = 62. The green line represents indels, splicing and nonsense mutations; blue line represents missense mutations

For mild mutations, hearing was normal before school age, hearing loss began at school age and gradually increased with increasing age (Fig. 4). For severe mutations, there was mild hearing loss at preschool age. Hearing loss gradually increases with age, and during the teenage years, it reaches about 50 dB HL and enters a platform period.

These results suggested a significant correlation between the degree of hearing and genotype.

#### The relationship between PTA’, age, mutation type and change of amino acid

**Fig. 5:**
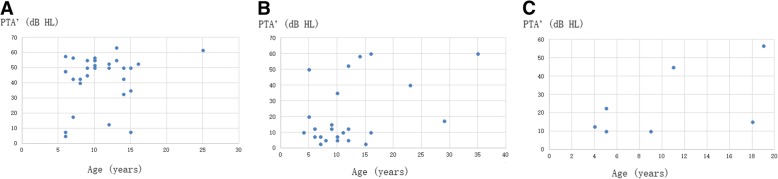
Relationship between PTA’, age, mutation type and change of amino acid. X-axis represents age and the Y-axis represents PTA’; **a** Indels, splicing and nonsense mutations (n = 31); **b** Replacement of glycine in missense mutations (*n* = 24); and **c** Replacement of non-glycine in missense mutations (*n* = 7)

The more serious the mutation type was, the greater the hearing loss was. However, normal hearing was still present in those patients with indels, splicing and nonsense mutations. For missense mutations, hearing was normal, but severe hearing loss was still present in these patients. Among patients with hearing loss, missense mutations were identified and glycine substitutions were the most common type (Fig. 5).

These findings suggested that the hearing level was associated with the severity of gene mutations and to the types of amino acid changes.

The relationship between hearing loss and distribution of missense mutation sites (along the gene) was presented in Table 3.

**Table 3:** Clinical data for hearing and distribution of mutation sites (missense mutations)

	Exons		Total mutations (n)
	1–25	26–51	
missense mutations			
Normal hearing (n)	4	19	23
Abnormal hearing (n)	3	6	9
Total (n)	7	25	32

Chi-squared test: P>0.05; not statistically significant (a *p* value of less than 0.05 was considered to be statistically significant).

There was no correlation between the hearing level and the distribution of mutation sites (along the gene).

### Factors that affect hearing

The possible factors that could influence hearing were age, variant type, Up/24 h, and renal function (Ccr). All the variables listed were analyzed using multiple regression analysis. The linear regression equation was as follows:

PTA’ = − 12.238 + 5.628*Renal function+ 18.458*Variant type.

The results showed a positive correlation between hearing and type of mutation and renal function. The more severe the type of mutation was, the worse the renal function was, and the poorer the hearing was.

## Discussion

There are three types of inheritance in AS: X-linked dominant inheritance, autosomal recessive and autosomal dominant inheritance. Among them, X-linked inheritance is the most important type of inheritance. An earlier study [27] showed that the XLAS patients have dominant genetic characteristics in both males and females. However, the hearing loss was not manifested in all XLAS patients [15].

This study showed that the hearing impairments of X-linked male patients were characterized as bilateral symmetrical sensorineural deafness, with an incidence of 63.2% (55/87), and was consistent with that of the literature [15]. The majority of patients presented with mild-to-moderate hearing loss, and 4 presented with moderate-to-severe hearing loss. In our study, hearing loss was usually started in the middle frequencies and gradually affected high frequencies, which was different from that in the paper [20]. Our results reported that hearing loss usually started at school age and gradually increased with increasing age. However, it maintained a relatively steady platform level of 50–60 dB HL during the teenage years. The shape of the audiometric curve was mainly groove-type (92.73%), which was quite different from the level reported in the literature [20]. The characteristics of hearing loss and hearing curve in this study are different from those reported in previous literatures, and this may be related to sample size or age distribution of the subjects. The characteristics of groove-type audiometric curve is clinically rare, contributing to the diagnosis of AS. This meant that, if the hearing loss plots as a groove-type audiometric curve, a preliminary diagnosis of AS can be made in children with hematuria or proteinuria before kidney puncture, skin biopsy pathology, or genetic diagnosis. Studies [28] have shown that the proportion of patients with AS misdiagnosed as other kidney diseases was up to 86%. Combined with the typical groove-type curve of hearing loss, a preliminary early diagnosis can be made. The audiological features and the relationship between hearing and genotype were analyzed as follows.

Hearing loss in male XLAS patients initially involved the middle frequency range and was gradually increased with increasing age. Middle frequency hearing was relatively stable when it had dropped to a certain level, and then high frequency hearing began to decline. It is speculated that this change in audiology is related to cochlear middle basement and basal membrane disease. During the early stages of the disease, damage to middle basement membrane was more obvious than to the basal membrane, and the lesion on the basal membrane was not sufficient to cause high-frequency hearing loss. With increasing time and age, the middle basement and basal membrane lesions gradually became more severe, with the development of hearing loss at higher frequencies. We hypothesized that there was a different expression and metabolism of type IV collagen regulatory genes in the middle and basal membrane of the cochlea, resulting in different manifestations of early middle frequency hearing loss.

*COL4A5* gene mutation is the cause of XLAS, and so far, more than 300 *COL4A5* mutation sites have been found [29]. All 87 patients in this study were tested for *COL4A5/6* genotype, and 60 different *COL4A5* pathogenic variants were identified in 63 patients. There are various forms of *COL4A5* gene mutations, including rearrangement, deletion, insertion and point mutations, leading to splicing mutations, frameshift mutations, missense mutations or truncation mutations. Mutations such as rearrangement, nonsense and frameshift mutations led to the abnormal structure or function of type IV collagen, and are also known to be severe mutations in AS [15, 16]. Our research concluded that missense mutations are less likely to lead to hearing loss than other mutations. In addition, this study also showed that the type of mutation affects the degree of hearing loss and the age of onset. The more severe the mutation type was, the earlier and the more serious was the hearing loss. The audiological phenotype and genotype are closely related. It has been reported that the risk of developing hearing loss before the age of 30 was about 60% in patients with missense mutations, whereas it reached 90% for the other types of mutations together including splice site mutations [16]. However, this phenomenon has not been observed in our study. The reasons for this might be due to that most of our outpatients are children, and the population included was basically children-only, where one was over 30-years-old. An earlier study showed that the location of the mutation site is also related to the time of occurrence of end-stage renal disease, i.e., the closer the mutation site is to the 5 ‘end of the gene, the earlier the occurrence of the disease [30]. However, in this study, there was no significant correlation between hearing level or the age of onset of hearing loss and mutation sites.

It has been reported that in patients with mild gene mutations, hearing loss remains mild [16]. However, in this study, 9 patients with missense mutations had moderate hearing loss, wherein 7 cases had glycine replaced by other amino acids, 1 case of proline replaced by serine, and 1 case of serine replaced by aspartic acid. Even if the amino acid changes are all from glycine to other amino acids, there was a significant difference in the secondary structure of the α5(IV) chain and the clinical phenotype of the kidney. This might be due to the fact that the glycine is located in different positions in the same domain of α5(IV). Moreover, the degree of change of secondary structure is consistent with the severity of the corresponding renal clinical phenotype [5]. The gene mutation in one patient in this study was a missense mutation in which the glycine was replaced by aspartic acid and the site was located in the exon 49. Glycine is polar and uncharged, whereas aspartic acid is a negatively polar charged molecule. So, it was speculated that the change in the charge properties of amino acids may affect the normal folding of whole α5(IV) chain. This affects the structure of the basement membrane, leading to a change in hearing. Therefore, it is thought that some missense mutations that caused glycine to be replaced by non-identical amino acids may not be mild mutations, but a more serious clinical phenotype [31–33].

The missense mutations in *COL4A5* collagen region, such as the replacement of glycine or arginine, usually result in a more severe disease in the kidney [34]. In this study, 5 patients with severe mutations had normal hearing, and their ages were 6, 6, 7, 12 and 15 years old, respectively. This cannot be explained by factors such as the severity of mutations and changes in age. The analysis found that the gene mutation sites were located in exons 8, 19, 22, 25, and 33, respectively, with the mutation site closer to the 5′ end. However, in this study, the relationship between hearing and the location of the mutation was statistically analyzed, and there was no obvious correlation between the hearing level and the location of the mutation, which was inconsistent with the previous reports [30]. Therefore, further studies are warranted.

At the same time, a total of 49 patients were followed up for more than 2 years, where 28 cases showed a decreasing trend in the hearing level of about 5 dB per year. Through observation, it was found that there was no significant difference in the degree of progression of hearing loss between patients with mild mutations and those with severe mutations after hearing loss had occurred. The European Union’s AS Collaborative Group conducted a study on this phenomenon to evaluate the genetic heterogeneity of diseases among different families of the disease and to study the relationship between genotype differences and end-stage renal disease progression. Therefore, it is necessary for us to further expand the sample size and follow-up time to study the relationship between the auditory genotypes and the phenotypes.

Through family studies, we identified the phenomenon of co-segregation of clinical phenotypes and genotypes. The pathogenicity of 17 kinds of gene mutations found in this study has already been reported in the previous studies. We conducted nucleic acid conservation and pathogenicity analysis and amino acid conservation analysis for unreported gene mutations in this study. The PhastCons and PhyloP software showed that the amino acid sequences were almost highly conserved. Polyphen-2 and SIFT gene prediction softwares were used to predict the possible pathogenicities of 20 unreported missense mutations. Polyphen-2 prediction software could not be able to predict the outcome in 2 patients. In one of these 2 cases, SIFT predicted the pathogenicity, while the other patient was non-pathogenic. The remaining 18 unreported missense mutations were likely to be pathogenic. The severe mutations were predicted to be pathogenic. Further cytological and animal experimental studies should be conducted for those novel mutations that discovered gene loci in this study.

## Conclusions

AS is a rare disease. We have conducted a preliminary study on the clinical audiological characteristics and the relationships between auditory genotypes and phenotypes in AS, and finally reached to certain conclusions. In conclusion, this study contributed to the timely diagnosis of the disease, and has important clinical implications in estimating the progression and prognosis of hearing loss. The limitations of the study include small sample size and short follow-up time. Our professional group will continue to collect the data to further study the correlation between auditory phenotypes and genotypes. This is a broad research field for future work, and genetically modified animals or animal models should be employed to understand the disease mechanisms involved in hearing loss.

## Abbreviations

AS: Alport syndrome
Ccr: Creatinine clearance rate
ESRD: End-stage renal disease
GERP: Genomic evolutionary rate profiling
OAE: Otoacoustic emissions
PCR: Polymerase chain reaction
PTA: Pure tone audiometry
RT-PCR: Reverse Transcription-Polymerase Chain Reaction
Up: Urinary protein
WHO: World Health Organization
XLAS: X-linked Alport syndrome
